# Few-Layered MoS_2_/Acetylene Black Composite as an Efficient Anode Material for Lithium-Ion Batteries

**DOI:** 10.1186/s11671-017-2322-3

**Published:** 2017-10-03

**Authors:** Rajashekar Badam, Prerna Joshi, Raman Vedarajan, Rajalakshmi Natarajan, Noriyoshi Matsumi

**Affiliations:** 1 School of Materials Science, Japan Advanced Institute of Science and Technology, Nomi, Ishikawa 923-1292 Japan; 2 Centre for Fuel Cell Technology, International Advanced Research Centre for Powder Metallurgy and New Materials (ARCI), IIT-M Research Park, Phase-1, 2nd Floor, Section B1, No. 6, Kanagam Road, Teramani, Chennai, 600 113 India

**Keywords:** Few-layered MoS_2_, Acetylene black, Anode material, Li-ion battery

## Abstract

Novel MoS_2_/acetylene black (AB) composite was developed using a single-step hydrothermal method. A systematic characterization revealed a few-layered, ultrathin MoS_2_ grown on the surface of AB. The inclusion of AB was found to increase the capacity of the composite and achieve discharging capacity of 1813 mAhg^−1^.

## Background

Lithium-ion battery (LiB) has been one of the most important rechargeable energy storage technologies used in a variety of portable electronic devices like laptops and cellular phones. In the present scenario of increasing pollution and fossil fuel depletion, the outlook in the research community for LiB power-sourced electric vehicles (EVs) has been increasing. The performance EVs is greatly dependent on the performance of the LiBs in terms of capacity and cyclability. Graphite, an extensively used anode material for commercial LiBs [1] offered a great deal of improvements compared to older carbonaceous and pure Li anode materials. But, the limited theoretical capacity of graphite (372 mAhg^−1^) asks for a replacement in order to increase the capacity of the LiB. There has been quite a lot of research concerning the improvement of anode materials to achieve high specific capacity and cyclability. After the inclusion of super material ‘graphene’ in LiBs, the achieved capacity was found to be twice that of graphite [2, 3]. While looking for other alternatives, MoS_2_ has been found to be a promising candidate with high capacity, ease of preparation, and low-volume expansion [4]. MoS_2_ is found to involve in four electron transfer reaction while hosting Li-ions enabling the capacity as high as 669 mAhg^−1^. Though the bulk MoS_2_ does not offer many exciting electrochemical properties for Li storage, the nanostructured counter parts of the same offers more exciting properties, hence collecting the large growing interest. Although there exist few nanostructured materials like carbon, silicon [5], tin [6], and tin dioxide [7], MoS_2_ outperforms these in terms of rate capacity and capacity retention along with economic viability. In spite of various prominent features, MoS_2_ as such cannot be employed as commercial anodic material in LiBs due to its low intrinsic electronic conductivity and large expansion and contraction after lithiation and delithiation resulting in pulverization and loss of electrical contact [8–10]. These in turn result in poor cyclability and dynamics of MoS_2_ lithium storage. Various strategies have been worked on to eliminate these disadvantages. They are MoS_2_ nanostructures with varied morphologies, MoS_2_/conducting polymer hybrids [1, 11], and MoS_2_/carbon nano composites [12–14]. Recently, MoS_2_/carbon materials were found to be appealing due to its capacities as high [15, 16] as 1000 to 1100 mAhg^−1^. The present research work deals with the preparation of novel few-layered MoS_2_/AB composite material using a wellknown but neglected carbon black, acetylene black, as the conducting substrate. Along with possessing high surface area to volume ratio, it exhibits interestingly very good interaction with the electrolytes [17]. It is also well known for its binding ability and used in preparing good inks to make carbon electrodes providing binding ability along with good electronic conductivity. The study demonstrates not only AB as a good conducting substrate but also as a better nucleation material for the preparation of highly crystalline few-layered MoS_2_. The systematic characterization and application of the material as anode in LiB lead to achieve ~ 1813 mAhg^−1^. To the best of our knowledge, this is the first time a few-layered MoS_2_/AB composite has been used as an anode material for achieving high specific capacity.

## Methods

### Synthesis

The few-layered MoS_2_/AB composite was prepared using facile single-pot hydrothermal method. In this, 1 mmol of Na_2_MoO_4_ 2H_2_O and 5 mmol of thiourea were dissolved in 60 ml of water to which 100 mg of AB was added. This solution was ultrasonicated for 30 min to get a homogeneous solution which was held at 210 °C for 24 h in an autoclave. The resultant material was filtered, washed thoroughly with water, and dried at 100 °C under vacuum for 12 h. Generally, along with the active material, a conducting carbon (acetylene black) will be used along with a polymer binder like polyvinylidene fluoride to make an ink. In the present study, AB is used as a conducting substrate and nucleation substrate to grow few-layered MoS_2_. So, the use of the extra 10% of conducting carbon is omitted in the ink preparation method. In brief, MoS_2_/AB composite along with 10 wt% of PVDF was taken into a beaker, to this isopropyl alcohol, and NMP mixture was added to disperse using ultrasonication. A uniformly dispersed slurry was prepared thus and then it was spray coated over a Cu foil. The electrode was dried at 120 °C under vacuum overnight to remove traces of solvents used. The electrode consisted of 0.728 mg/cm^2^ of active material.

### Material Characterizations

The diffraction pattern of the material was characterized by a powder X-ray diffraction technique (Smart Lab X-Ray Diffractometer, Rigaku) with Cu Kα radiation (λ = 0.154 nm, over the 2θ range of 10°–70° with a step size of 0.005°). Elemental analysis of the material was studied using X-ray photoelectron spectroscopy (XPS) technique performed on S-Probe™ 2803 instrument. The morphological characterization was done using high-resolution transmission electron microscopy (HR-TEM) on Hitachi H-7650 model. TEM sample was prepared by dropping a methanolic solution containing a well-dispersed composite on a carbon-coated copper grid.

## Results and Discussion

**Fig. 1:**
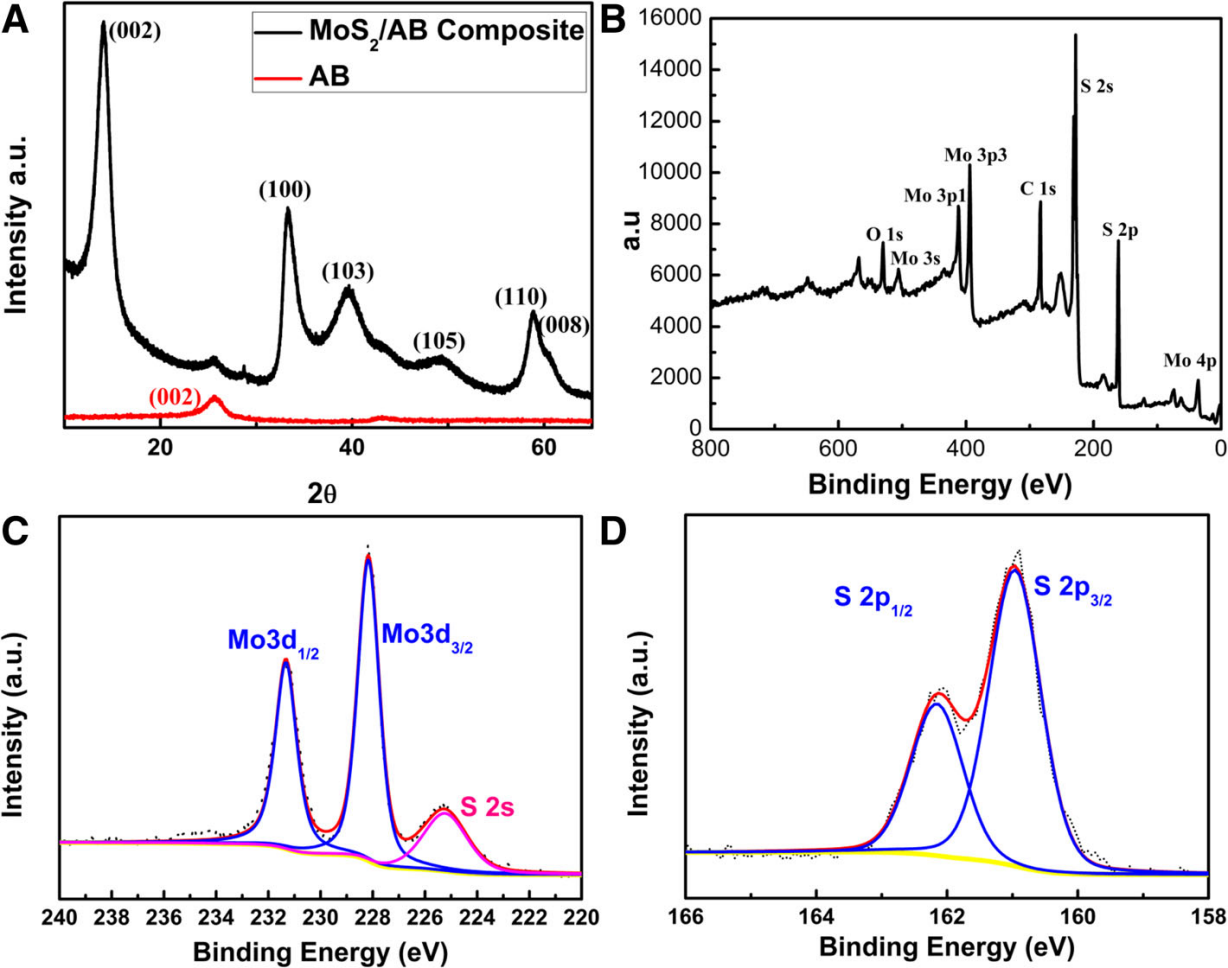
(**a**) XRD diffraction patterns for MoS_2_/AB and raw AB. (**b**) XPS survey spectrum showing the presence of Mo, S, C, and O. **c** High-resolution spectra of Mo 3d peak and **d** S 2p

**Fig. 2:**
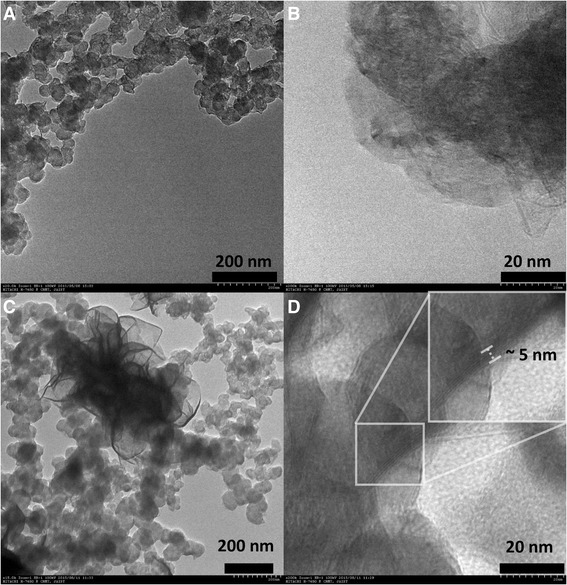
TEM micrographs of **a** AB showing interconnected morphology. **b** Highly layered nature of AB. **c** MoS_2_ nanocrystal showing flower-like morphology. **d** Layered and transparent morphology of MoS_2_ with inset showing a close view of few layers of MoS_2_ in the prepared composite

The diffraction pattern of the as prepared material is shown in Fig. 1a. The weak and broadened peak at 25° to 28° can be assigned to the carbon (002). All the other sharp and distinctive peaks imply the phase purity and high crystallinity. These peaks agree well with the hexagonal MoS_2_ planes [2]. The average *c*-stacking height was calculated using Scherrer equation (*kλ*/*β*cos*θ*) where *k* is the shape factor, *λ* is the X-ray wavelength, *β* is the full width at half maxima (FWHM) of (002) peak of MoS_2_, and *θ* is the peak position. The calculated *c*-stacking height was found to be 5 nm consisting five to six layers of MoS_2_. Elemental analysis of MoS_2_/AB composite was performed using XPS. Sampling for XPS was carried out by drop casting the material on Cu foil. The XPS survey spectrum (Fig 1b) indicated the presence of Mo, S, C, and a small amount of O. The Mo:S ratio corroborated stoichiometric of the material to be in the form of MoS_2_. The composition also confirmed the MoS_2_:AB as 8:2; in other words, the amount of AB in the composite was 20%. Deconvolution of high-resolution peak (Fig 1c) of molybdenum revealed two peaks at 231.3 and 228.2 eV corresponding to 3d_3/2_ at 3d_1/2_ of Mo(IV). The absence of any other 3d peak confirms the absence of other higher oxidation states of Mo; in other words, the absence of oxide of Mo was evident. The oxygen atom percentage in the MoS_2_/AB and AB is found to be same. Hence, this oxygen can be attributed solely to the functional moieties of AB, which in turn reiterates the absence of oxide forms of Mo. The sulfide peaks at 161 and 162.1 eV represents the 3p_1/2_ and 3p_3/2_ of sulfide (Fig 1d). This confirms the presence of pure MoS_2_ in the composite. The morphological features of the MoS_2_/AB composite were studied using TEM. Figure 2a shows the highly interconnected morphology of AB, and Fig. 2b shows the finely layered AB and graphene like transparent 2D layers are exposed at the edges. TEM micrographs (Fig. 2c, d) shows that the MoS_2_/AB is of layered nature with a few layers of MoS_2_ along with the interconnected nature of AB running all along as a connecting thread. The morphology of the MoS_2_ layers are found to be a flower-like structure, and the particulate size was found to be around 650 nm distributed on a network of AB. With this, it is very clear that the aspect ratio of MoS_2_ with respect to the particle size of AB is very high which makes AB to be a very good conducting linker. The high surface area to volume ratio of the AB makes it a uniform conducting network distributed across the MoS_2_ matrix even with just 20 wt%. Figure 2d also corroborates the similar results as XRD showing five to six layers of MoS_2_. As AB was found to be having a lot of structural and functional defects, the enhanced nucleation can be expected to form a uniform and more dispersed layers having AB as conducting a back bone. The presence of AB also greatly restricts the few-layered nature of MoS_2_ from restacking.

**Fig. 3:**
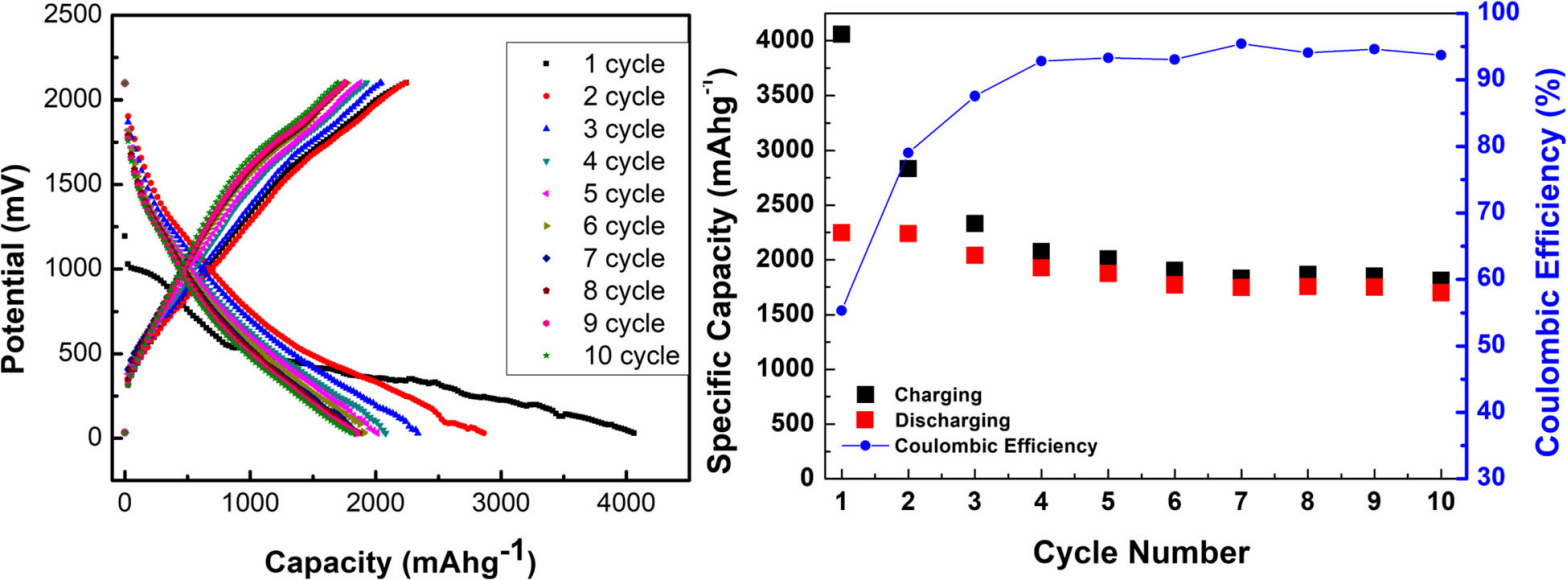
**a** Charge discharge curves for 10 cycles (left) and **b** coulombic efficiency and specific capacity vs cycle number (right)

To understand the electrochemical performance of the few-layered MoS_2_/AB composite, especially to study the effect of highly exfoliated MoS_2_ and AB to the capacity, galvanostatic charge-discharge tests in a conventional coin cell type were performed. The half cell was made using Li foil as cathode and as prepared MoS_2_/AB composite as anode separated by a celgard® separator, and 0.1-M LiTFSI salt in EC:DEC was used as electrolyte. Charge discharge was done at a 0.090-mA rate between 2.10 and 0.03 V. Figure 3a shows the charge discharge curves for the same. The typical charge discharge curves were observed in the case of MoS_2_/AB composite. The plateau at around 1.0 V vs Li/Li^+^ represents the insertion of lithium.

$$ Mo{S}_2+ xL{i}^{+}+x{e}^{-}\to L{i}_x Mo{S}_2 $$

The lower plateau occurring at 0.6 V vs Li/Li^+^ can be attributed to the conversion reaction process, which first entails either the reversible in-situ decomposition of MoS_2_ into metallic Mo embedded into a Li_2_S matrix and/or resulting in the gel-like polymer layer from the electro-degradation of electrolyte. Both these potential plateau disappear after the first few cycles. In the delithiation curve, a small but conspicuous plateau is seen at around 1.7 V, and it is typical for highly crystalline phase of MoS_2_ in the composite. As shown in Fig. 3a, the composite gave enormously high first lithiation capacity of 4086 mAhg^−1^, and then, the reversible capacity was found to be around 1813 mAhg^−1^. Figure 3b shows coulombic efficiency and specific capacity vs number of cycles. Following its first cycle, the MoS_2_/AB composite electrode showed a reversible charge/discharge behavior, exhibiting a stable capacity of about 1813 mAhg^−1^ with coulombic efficiency of > 95%. The reversible capacity of the present material is found to be 523 mAhg^−1^ higher than that of the championship material till date. The enhanced capacities can be attributed to the following factors: (i) highly exfoliated and few-layered MoS_2_, (ii) synergistic effect between few-layered MoS_2_ and layered AB, (iii) improved lithiation and delithiation due to the presence of AB with high absorptivity of electrolyte, and (iv) improved electronic conductivity with the introduction of AB.

## Conclusions

In conclusion, the present research work includes the preparation of novel few-layered MoS_2_/acetylene black composite material in a very simple hydrothermal method. An as prepared material was systematically characterized to understand the morphology and chemical compositions. The material was fabricated as an electrode and charge/discharge characterizations in a LIB anodic half cell was carried to elucidate the capacity behavior of the same. The studies were conducted in a conventional coin cell setup using a half-cell configuration. The obtained results envisage the composite material prepared as a promising candidate as an efficient anode material for LIBs. To the best of our knowledge, the achieved capacities of 1813 mAhg^−1^ with the composite are leading the contingent of all the other MoS_2_-family-based materials till date.
